# Magnetic resonance imaging in leukodystrophies: characteristic patterns and diagnostic relevance

**DOI:** 10.1055/s-0046-1824758

**Published:** 2026-07-17

**Authors:** Matheus de Lima Ruffini, Danilo de Assis Pereira, Rafael Saliba Helmer, Adolfo Moraes de Souza, Bernardo Savaya Lima, Gabrielle Baccarin, Gustavo Kazuo Yamada, Luiz Fernando Monte Borella, Renan Denadai Turci, Juliana Ávila Duarte, Fabiano Reis

**Affiliations:** 1 Universidade Federal do Rio Grande do Sul, Faculdade de Medicina, Departamento de Radiologia e Oncologia, Porto Alegre RS, Brazil.; 2 Pontifícia Universidade Católica de São Paulo, São Paulo SP, Brazil.; 3 Universidade Estadual de Campinas, Faculdade de Ciências Médicas, Campinas SP, Brazil.

**Keywords:** Alexander Disease, Magnetic Resonance Imaging, Demyelinating Diseases, Nervous System Diseases, Pediatrics, Neuroimaging

## Abstract

**Background:**

Leukodystrophies are inherited disorders that primarily affect the central nervous system white matter and often present with nonspecific symptoms, making early diagnosis difficult. Magnetic resonance imaging (MRI) is the key initial imaging test because it reveals characteristic myelin patterns that can guide biochemical and genetic workups.

**Objective:**

To review clinically useful MRI and MR spectroscopy (MRS) patterns in major leukodystrophies and emphasize their diagnostic relevance using a practical, pattern-recognition approach.

**Methods:**

Narrative review of the literature integrating conventional MRI, including T1/T2 fluid-attenuated inversion recovery (FLAIR), and diffusion-weighted imaging (DWI), as well as advanced techniques (MRS), with a pictorial, case-based approach.

**Results:**

The pivotal imaging distinction is between hypomyelination—diffuse, persistent T2/FLAIR hyperintensity with relative temporal stability and absent enhancement—and demyelination, characterized by confluent, progressive lesions with disorder-specific topographic predilection. Recognizable signatures include U-fiber sparing and a tigroid pattern in metachromatic leukodystrophy (MLD); parieto-occipital predominance with trizonal enhancement and restricted diffusion in X-linked adrenoleukodystrophy (X-ALD); frontal predominance and the “tadpole sign” in Alexander's disease; optic pathway and thalamic involvement in Krabbe's disease; markedly elevated N-acetylaspartate (NAA) on MRS in Canavan's disease; and tract-selective leukoencephalopathy brainstem/spinal cord involvement with a lactate peak (LBSL). These imaging clues refine differential diagnosis, guide targeted genetic testing, and support longitudinal monitoring.

**Conclusion:**

The use of MRI—especially when complemented by DWI and MRS—remains central for early recognition and classification of leukodystrophies. A structured, pattern-based approach integrating clinical context with imaging topography can reduce diagnostic delay and support timely management.

## INTRODUCTION

Leukodystrophies constitute a heterogeneous family of inherited disorders that principally affect central nervous system white matter, classically manifesting in childhood but also occurring in adults, in whom clinical and imaging features can differ from pediatric forms. 1 Because initial symptoms are often nonspecific, magnetic resonance imaging (MRI) is the diagnostic cornerstone: it uncovers early or presymptomatic myelin abnormalities and displays signatures—symmetrical lesions, U-fiber sparing, cystic change, deep gray involvement—that steer biochemical and molecular testing toward the correct genetic diagnosis ( Table 1 ). 1

**Table 1:** Important tips for diagnosis

Clinical manifestation	Often nonspecific; clinical history, age of onset, and measurements such as head circumference (e.g., macrocephaly) should be considered.
Comprehensive diagnosis	Requires biochemical, enzymatic, and molecular tests, plus metabolic analysis.
Monitoring	Essential for tracking disease progression and treatment response.
Role of magnetic resonance imaging	Key for identifying white matter abnormalities and distinct MRI patterns specific to different leukodystrophies.

**Table 2:** Clinicoradiological approach to leukodystrophies: age stratification, key clinical features, MRI signatures, and differential diagnosis

Disease	Age	Clinical	MRI	Differential diagnosis
PMD	Infancy (clinical spectrum from connatal to milder phenotypes)	Pendular nystagmus, axial hypotonia with subsequent spasticity, developmental delay, ataxia, and progressive cognitive impairment	Diffuse hypomyelination with persistently high T2/FLAIR signal in cerebral white matter; corpus callosum thinning; absence of contrast enhancement and diffusion restriction; MRS may show mildly reduced NAA and increased choline and creatine	Other hypomyelinating leukodystrophies (Deletion 18q syndrome)
Deletion 18q syndrome	Childhood	Developmental delay, hypotonia, intellectual disability, seizures, craniofacial dysmorphism, hearing loss, and growth retardation	Symmetric hypomyelination with diffuse periventricular T2/FLAIR hyperintensity and thin or hypoplastic corpus callosum; MRS may show mildly reduced NAA and slightly increased choline; α-glutamate elevation has been reported	Other causes of hypomyelination associated with syndromic neurodevelopmental disorders (PMD)
MLD	Pediatric onset is typical	Progressive motor and cognitive decline	Confluent, symmetric T2/FLAIR hyperintensity in cerebral white matter with radial hypointense streaks; relative sparing of subcortical U-fibers; cranial nerve or cauda equina enhancement may occur early; DWI may show signal increase in active zones; progressive atrophy may develop	Other demyelinating leukodystrophies (Krabbe disease is one of the differential diagnosis)
X-ALD	Childhood cerebral phenotype typically 4–10 years; frontal presentations also described	Behavioral changes, cognitive decline, sensorineural deficits, and adrenal insufficiency	Symmetric parieto-occipital T2/FLAIR hyperintensity involving splenium and corticospinal tracts; peripheral rim enhancement consistent with active inflammatory demyelination (trizonal pattern); DWI restriction in active lesions; MRS commonly shows reduced NAA and elevated choline	Other inflammatory demyelinating and inherited white matter disorders in childhood (acyl-coenzyme A oxidase deﬁciency)
MLC	Childhood	Progressive macrocephaly with variable motor impairment	Megalencephaly with diffuse T2/FLAIR hyperintensity of subcortical and deep white matter; characteristic subcortical cysts, classically in temporal poles; persistent cavum septum pellucidum may be present; diffusion metrics often show increased ADC and reduced fractional anisotropy; MRS may show increased choline and myoinositol with slight NAA reduction	Other macrocephaly-associated leukoencephalopathies
Alexander's disease	Infantile form typically <2 years; juvenile/adult forms have slower progression	Infantile: macrocephaly, regression, seizures, spasticity, bulbar dysfunction; juvenile/adult: bulbar signs, cerebellar ataxia, pseudobulbar affect	Frontal-predominant T2/FLAIR hyperintensity affecting periventricular and subcortical white matter; frontal atrophy; basal ganglia signal changes; T1 hyperintense periventricular rim; frequent contrast enhancement in periventricular regions and brainstem; spinal cord enlargement early with subsequent atrophy	Other frontal-predominant leukodystrophies (i.e. frontal variant of X-ALD)
Krabbe disease	Infantile form usually within first 6 months	Irritability, hypertonia, developmental regression, feeding difficulties, and severe peripheral neuropathy	Early cerebellar involvement with characteristic dentate region signal pattern; thalamic T2 hypointensity; optic nerve and chiasm enlargement; cranial nerve or cauda equina enhancement may occur; progressive periventricular and centrum semiovale involvement with later corpus callosum and parieto-occipital abnormalities; diffusion changes may vary with stage	MLD and X-ALD
Canavan disease	Typically, 3–6 months	Macrocephaly, axial hypotonia, poor head control, developmental regression, seizures, and visual impairment	Bilateral, symmetric T2/FLAIR hyperintensity predominantly affecting subcortical white matter with early deep gray involvement; absence of U-fiber sparing and contrast enhancement; DWI may show reduced ADC in affected white matter; MRS shows markedly elevated NAA	Other infantile-onset macrocephaly-associated leukoencephalopathies
LBSL	Childhood or adolescence	Slowly progressive cerebellar ataxia, spasticity, and dorsal column dysfunction; cognition often relatively preserved	Symmetric T2/FLAIR abnormalities in periventricular cerebral white matter with tract-selective involvement of brainstem and spinal cord (including pyramids, cerebral peduncles, and posterior/lateral columns); MRS demonstrates lactate peak	Other mitochondrial leukoencephalopathies
VWM	Typically, 2–6 years; earlier onset correlates with more rapid progression	Progressive ataxia and spasticity with stress-provoked neurological deterioration; seizures and cognitive decline may occur; ovarian failure may be present in females	Diffuse T2 hyperintensity with progressive rarefaction and cystic degeneration, evolving toward CSF-like signal intensity; corpus callosum thinning with signal alteration; characteristic stripe-like sagittal appearance; absence of enhancement and diffusion restriction, with facilitated diffusion in cavitated regions	Other rarefying and cystic leukoencephalopathies
POLR3-related leukodystrophies	Childhood	Motor delay, gait disturbance, cerebellar ataxia, spasticity; hypodontia, short stature, and hypogonadotropic hypogonadism are important extra-neurological clues	Diffuse symmetric hypomyelination involving cerebral white matter and cerebellum; involvement of posterior limbs of the internal capsule and corticospinal tracts; thin but preserved corpus callosum; early vermian atrophy; characteristic T2 hypointensity of optic radiations and anterolateral thalami; no enhancement	Other hypomyelinating leukodystrophies
ALSP	Typically, 30–50 years	Neuropsychiatric symptoms with progressive cognitive decline, followed by parkinsonism, spasticity, and gait impairment	Bilateral, often asymmetric frontal and parietal T2/FLAIR white matter hyperintensity; early corpus callosum thinning and signal change; progressive frontal atrophy; foci of diffusion restriction may occur; CT may show punctate calcifications; MRS may show reduced NAA and elevated choline	Other adult-onset genetic leukoencephalopathies with callosal involvement
Glutaric aciduria (pattern described for L-2-hydroxyglutaric aciduria)	Childhood	Seizures, developmental delay, intellectual disability, movement disorders, and tone abnormalities	Predominant subcortical white matter involvement with relative sparing of periventricular white matter; globus pallidus and dentate nucleus T2/FLAIR hyperintensity; diffusion features consistent with increased diffusivity in chronic disease; MRS may show elevated choline and occasional lactate	Other metabolic leukoencephalopathies
NCL	Subtype-dependent, often childhood	Epilepsy, developmental regression, cognitive and motor decline, and progressive visual impairment	Thalamic T2/FLAIR hypointensity; hyperintensity in periventricular and deep white matter, posterior limb of internal capsule, ventral pons, and insular regions; supra- and infratentorial atrophy with progression on follow-up	Other neurodegenerative and metabolic leukoencephalopathies
KSS	Before 20 years	Progressive external ophthalmoplegia and pigmentary retinopathy; cardiac conduction block; cerebellar ataxia; sensorineural hearing loss; short stature and endocrinopathy	Cerebral and cerebellar atrophy with bilateral symmetric T2 hyperintensities in subcortical cerebral and cerebellar white matter, mediodorsal thalamus, basal ganglia, corpus callosum, and dorsal brainstem; spinal cord involvement is common	Other mitochondrial diseases

Abbreviations: ADC, apparent diffusion coefficient; ALSP, adult-onset leukoencephalopathy with axonal spheroids and pigmented glia; CSF, cerebral spinal fluid; DWI, diffusion-weighted imaging; FLAIR, fluid attenuated inversion recovery; KSS, Kearns–Sayre syndrome; LBSL, leukoencephalopathy with brainstem and spinal cord involvement and lactate elevation; MLC, megalencephalic leukoencephalopathy with subcortical cysts; MLD, metachromatic leukodystrophy; MRI, magnetic resonance imaging; MRS, MR spectroscopy; NAA, N-acetylaspartate; NCLs, neuronal ceroid lipofuscinoses; PMD, Pelizaeus–Merzbacher disease; VWM, vanishing white matter disease; X-ALD, X-linked adrenoleukodystrophy

Imaging techniques are equally critical for longitudinal care, enabling clinicians to distinguish static from rapidly progressive phenotypes, quantify lesion burden, and monitor therapeutic response. 2 Serial MRI every 6 to 12 months can refine prognosis and inform management decisions, including the timing of hematopoietic stem-cell transplantation and inclusion in gene-therapy trials. 3

Advanced MRI techniques can complement conventional imaging by providing quantitative, tissue-level biomarkers that help characterize leukodystrophy pathology and monitor disease burden. Examples include T1 mapping, which can reflect changes in myelin and water content, and diffusion tensor imaging, which can depict tract-specific involvement and quantify microstructural damage (both features that may support pattern recognition and follow-up in leukodystrophies). Quantitative MRI techniques may be advantageous by providing more specific and quantitative insight into different pathologies at tissue level. 2

In diffusion tensor imaging, changes in fractional anisotropy (FA), as well as mean (MD), axial (AD), and radial (RD) diffusivity, suggest that leukodystrophies affect aspects of brain microstructure. These changes most likely reflect a multitude of pathological processes, such as accumulation of specific substances, followed by myelin and axonal loss. The differences between untreated and treated patients indicate that diffusion measures are positively affected by hematopoietic cell transplantation, further emphasizing the beneficial effects of this intervention on white matter and supporting the findings of other quantitative MR measures. These measures provide more insight into time-dependent disease mechanisms and may help determine the optimal window for intervention. 4

Among these techniques, quantitative susceptibility mapping (QSM) may add value by probing susceptibility sources related to neuropathology in leukodystrophies, such as calcification, iron accumulation, or microbleeds, though the literature on its application in leukodystrophies is limited. One report on leukoencephalopathy with calcifications and cysts 5 demonstrated that QSM is able to identify typical parenchymal changes, comprising calcifications and microcalcifications, as well as ferritin deposits. These tissue modifications are caused by microangiopathy, which is thought to be the underlying mechanism leading to leukoencephalopathy, cyst development, and degenerative parenchymal calcifications. In cases of suspected microhemorrhage or diffuse parenchymal calcification, QSM could be added to the protocol.

This review and case series aim to provide an educational and up-to-date overview of characteristic MRI patterns in major leukodystrophies, correlating neuroimaging findings with underlying pathophysiological mechanisms, and offering practical insights through case examples to support diagnostic accuracy, differential diagnosis, and clinical decision-making.

## METHODS

### Subjects

This study included patients recruited from tertiary and quaternary referral centers in Brazil. All participants had a genetically confirmed diagnosis, with clinical and imaging features consistent with the respective diagnosis. Patients' ages ranged from 3 to 18 years, and both male and female participants were included.

### Imaging protocols

Imaging was performed using 1.5T or 3T MRI scanners, employing several sequences to assess various aspects of the brain's structure and white matter integrity. Furthermore, T1-weighted (T1W) scans were used to evaluate the overall structural integrity of the brain, aiding in the differentiation between gray and white matter. Also, T2-weighted (T2W) scans were utilized to detect abnormalities in the white matter, specifically focusing on hyperintensities that indicate areas of damage or disruption. To enhance the visibility of white matter abnormalities, particularly in the periventricular and subcortical regions, fluid-attenuated inversion recovery (FLAIR) imaging was employed. We acquired single- or multi-voxel ^1^ proton magnetic resonance spectroscopy (H-MRS), using a point-resolved spectroscopy (PRESS) sequence with short (30–40 ms), intermediate (135 ms), and long TE (270 ms), with localized shimming. The imaging protocol included:

- T1-weighted spin-echo (TR = 600 ms, TE = 10 ms, slice thickness = 1 mm);
- T2-weighted fast spin-echo (TR = 4000 ms, TE = 100 ms, slice thickness = 1 mm);
- FLAIR (TR = 9000 ms, TE = 120 ms, TI = 2500 ms, slice thickness = 1 mm);
- Magnetic resonance spectroscopy (MRS).

### Image analysis

The images were independently reviewed by two experienced neuroradiologists, who conducted a visual analysis focusing on key features. These included identifying white matter abnormalities, such as hyper- and hypointensities on T2-weighted and FLAIR images, and metabolic abnormalities on MRS. The analysis also focused on cortical and subcortical changes, with particular attention to cortical thinning, especially in the frontal and temporal regions, and involvement of the basal ganglia and thalamus. Additionally, special emphasis was placed on identifying patterns of white matter tract involvement, particularly within the corticospinal tract, optic radiation, corpus callosum, and other major pathways.

## RESULTS

On MRI the pivotal distinction is between hypomyelination, in which myelin fails to form, and demyelination, in which previously laid-down myelin is destroyed. 1 Hypomyelinating disorders show a diffuse lack of myelination, often observed as low T1 signal regions typically involving the internal capsule, corona radiata, and the optic radiation. On T2- and FLAIR-weighted images, there is an absence of the expected hypointensity in the supratentorial white matter, and the white matter volume is often reduced, with no contrast enhancement being seen. 1 3

Demyelinating forms show confluent, progressive lesions, and their topography is often diagnostic—periventricular or centrum semiovale in Krabbe and MLD, frontal in Alexander disease, subcortical in glutaric aciduria and Canavan disease, and parieto-occipital in X-ALD, as illustrated in the **Supplementary Material Figure S1** ( https://www.arquivosdeneuropsiquiatria.org/wp-content/uploads/2026/03/ANP-2025.0350-Supplementary-Material.docx ) and discussed throughout this section. Rim enhancement, calcifications, restricted diffusion, or cranial-nerve and thalamic signals sharpen the differential diagnosis and track disease activity. 1 3

**Figure 1:**
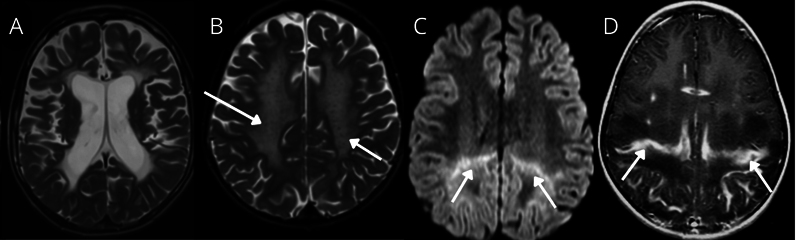
Abbreviations: DWI, diffusion-weighted imaging; MLD, metachromatic leukodystrophy; X-ALD, X-linked adrenoleukodystrophy. Magnetic resonance imaging (MRI) patterns in demyelinating leukodystrophies: MLD and X-ALD. ( **A** , **B** ) MRI in MLD. Axial T2/FLAIR images show diffuse, bilateral confluent hyperintensity involving the periventricular and subcortical white matter, with relative preservation of the U-fibers. The abnormalities are more pronounced in the frontal, parietal, and occipital lobes. On T2-weighted images, hypointense radial streaks are noted, consistent with a “tigroid” pattern. ( **C** , **D** ) X-ALD. ( **C** ) Axial DWI demonstrates restricted diffusion in the posterior periventricular white matter (arrows). ( **D** ) Post-contrast axial T1-weighted MRI shows the characteristic trizonal pattern: central hypointensity (necrosis), peripheral rim enhancement (active inflammation, arrow), and an outer hypointense zone corresponding to demyelination.

### Pelizaeus–Merzbacher disease (PMD)

Pelizaeus–Merzbacher disease (PMD) is a hypomyelinating leukodystrophy caused by mutations in the PLP1 gene, which encodes proteolipid protein 1, a major structural component of central neural system (CNS) myelin. 6 The disease usually presents in infancy with pendular nystagmus, axial hypotonia evolving into spasticity, developmental delay, ataxia, and progressive cognitive decline. The clinical spectrum ranges from the severe connatal form with neonatal onset to milder presentations such as spastic paraplegia type 2. 7

On MRI, PMD shows diffuse hypomyelination: the cerebral white matter remains hyperintense on T2/FLAIR and fails to acquire the age-appropriate hypointensity seen in normally myelinated tracts, often in combination with hypointensity on T1-weighted images. 8 The pontine abnormalities either consist of a global hyperintensity on T2-weighted images, often associated with hypointensity on T1-weighted images, or of T2 hyperintensity and T1 hypointensity confined to the pyramidal tracts in the ventral pons. The corpus callosum is frequently thin, and there is no contrast enhancement or diffusion restriction. The use of MRS may reveal mild reductions in N-acetylaspartate (NAA) and increases in choline (particularly in the frontal lobe and the centrum semiovale) and creatine. 9 However, increased levels of NAA in white matter have been reported in the literature. 10 11

Increased astrocytic processes, determined by staining for glial fibrillary acidic protein (GFAP), have been observed in PMD. These processes may be responsible for increased levels of choline and creatine, since in vitro spectroscopic data from primary astrocyte cultures has revealed both concentrations can be twofold higher than those in neuronal cell types. 12 As such, PMD remains the prototypical hypomyelinating disorder and illustrates the distinction between hypomyelination, demyelination, and rarefaction within the broader group of leukodystrophies. 13

### 18q deletion syndrome

The 18q-deletion syndrome results from terminal or interstitial loss of material on chromosome 18q—most often 18q21-q23—and the clinical severity parallels the size of the deleted segment. 14 Brain MRI typically shows symmetric hypomyelination, with diffuse T2/FLAIR hyperintensity of periventricular white matter and a thin or hypoplastic corpus callosum, while 1H-MRS may demonstrate mildly reduced NAA and slightly elevated choline, findings that reflect chronic axonal or myelin dysfunction rather than active demyelination; 15 it may also demonstrate elevated white matter α-glutamate (at 3.75 ppm). 16

Although these radiological features are not pathognomonic, when they coexist with the characteristic constellation of developmental delay, hypotonia, intellectual disability, seizures, craniofacial dysmorphism, hearing loss, and growth retardation, they should prompt cytogenetic testing; karyotyping or chromosomal microarray confirms the diagnosis by delineating the 18q deletion. 14 17 No disease-modifying therapy is available, so care is multidisciplinary and supportive.

### Metachromatic leukodystrophy (MLD)

Metachromatic leukodystrophy (MLD) is an autosomal-recessive lysosomal disorder caused by ARSA mutations that abolish arylsulfatase-A activity, allowing sulfatide accumulation and provoking demyelination within oligodendrocytes and Schwann cells, yielding the metachromatic reaction. 18 19 Analysis of MRI is striking, with confluent, symmetric T2/FLAIR hyperintensities in frontal, parietal and occipital white matter—the classic “white-as-snow” pattern—accompanied by radial hypointense streaks that mirror sulfatide deposition and, crucially, by relative sparing of subcortical U-fibers, a feature that helps distinguish MLD from other leukodystrophies ( Figure 1 ). 19 20 Cranial nerve or cauda equina enhancement may occur and precede white matter hyperintensities, providing a relevant early diagnostic clue and suggesting that blood-nerve barrier breakdown might be an initial event.

Diffusion-weighted imaging (DWI) in the active stage of the disease usually demonstrate moderate hyperintensity in the presumed progression zones of the disease process. The apparent diffusion coefficient (ADC) map data, however, indicates that T2-shine-through effect may also occur.

As the disease advances these lesions extend and global cerebral atrophy ensues, while clinically patients develop progressive motor and cognitive decline and ventriculomegaly. 19

### X-linked adrenoleukodystrophy (X-ALD)

The X-linked adrenoleukodystrophy (X-ALD) arises from pathogenic ABCD1 variants that cripple a peroxisomal very-long-chain fatty acid (VLCFA) transporter, leading to VLCFA accumulation, neuroinflammation, and demyelination. 21 22 The cerebral childhood phenotype—onset between 4 and 10-years-old—progresses rapidly with behavioral change, cognitive decline, sensorineural deficits and adrenal failure. 21 22 23 The signs in MRI are distinctive, showing symmetrical T2/FLAIR hyperintensity in parieto-occipital white matter, splenium of the corpus callosum, and corticospinal tracts. However, Loes et al. studied 206 males with X-ALD and found that 15.5% of the patients had an initial frontal lobe involvement (aged 10–16 years). 24

Rim enhancement at lesion margins produces the classic “trizonal” appearance, while DWI demonstrates restricted diffusion in active plaques, and MRS reveals reduced NAA with elevated choline ( Figure 1 ). 21 22

Isolated corticospinal tract or cerebellar white matter involvement is observed in a minority of patients and represents more slowly progressive involvement. Diagnosis is confirmed by elevated plasma VLCFAs and ABCD1 sequencing, and presymptomatic hematopoietic stem-cell transplantation can arrest progression. 21 22 25

### Megalencephalic leukoencephalopathy with subcortical cysts (MLC)

**Figure 2:**
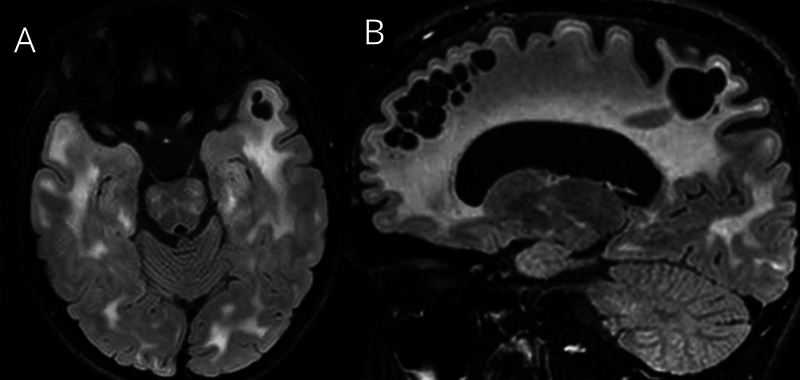
Abbreviations: MLC, megalencephalic leukoencephalopathy with subcortical cysts. Magnetic resonance imaging (MRI) features of MLC and Alexander's disease. ( **A** , **B** ) MLC. Axial ( **A** ) and Sagittal ( **B** ) T2/FLAIR images demonstrate diffuse, bilateral hyperintensity involving the deep and subcortical white matter, including the U-fibers, with frontal and temporal predominance. Subcortical cysts were identified (round hypointensities), particularly in the parietal and temporal regions ( **A** ) and in the frontal lobes ( **B** ).

Megalencephalic leukoencephalopathy with subcortical cysts (MLC) is usually autosomal-recessive due to MLC1 mutations, 26 and, less often, GLIALCAM or C-type natriuretic peptide (CNP) variants. It presents radiologically with striking megalencephaly and diffusely increased T2/FLAIR signal in both subcortical and deep white matter. 27 28 Its signature finding is thin-walled subcortical cysts, most conspicuous in the temporal poles but also seen in parietal and occipital lobes, accompanied by a persistent cavum septum pellucidum ( Figure 2 ); T1-weighted images show diffuse hypointensity of the affected white matter, and the absence of cerebellar abnormalities helps distinguish MLC from congenital muscular dystrophies in macrocephalic patients.

The use of DWI shows prominent hypointensity in the subcortical cysts and hypointensity in the affected white matter. The ADC is increased and diffusion tensor imaging data show reduced fractional anisotropy. Proton MRS typically demonstrates elevated choline and myoinositol with slightly reduced NAA—markers of gliosis and rarefaction—and, in advanced cystic stages, lactate and glucose peaks that indicate severe metabolic derangement. Clinically, children exhibit progressive macrocephaly but often maintain motor milestones for several years; the clinical course complements the MRI pattern and guides genetic confirmation.

### Alexander's disease

Alexander's disease is a progressive leukodystrophy produced by dominant glial fibrillary acidic protein (GFAP) gain-of-function mutations that generate astrocytic Rosenthal fibers and drive widespread white matter degeneration. 29 30 The infantile form is the most severe, manifesting within 2-years with megalencephaly, psychomotor regression, seizures, spasticity, and bulbar dysfunction, whereas juvenile and adult forms progress more slowly with bulbar signs, cerebellar ataxia, and pseudobulbar affect. 29 30 31 32 33

Analysis of MRI scans is distinctive: frontal-predominant T2/FLAIR hyperintensity involving periventricular and subcortical white matter, frontal atrophy, basal-ganglia signal change, and a characteristic T1 hyperintense periventricular rim; intense contrast enhancement frequently lines the ventricles and appears in basal ganglia, brainstem and middle cerebellar peduncles, while early spinal cord swelling and later spinal cord atrophy underscore the disease's rostrocaudal extension. 31 These imaging clues, together with GFAP sequencing, secure the diagnosis; treatment remains supportive.

### Krabbe's disease

Krabbe's disease is an autosomal-recessive lysosomal disorder caused by GALC mutations that abolish galactocerebrosidase activity; psychosine then accumulates and precipitates fulminant demyelination of both central and peripheral nervous systems. 34 The infantile form appears within the first 6 months with irritability, hypertonia, developmental regression, feeding difficulty and severe neuropathy, typically culminating in death before 2-years-old. 34 35

**Figure 3:**
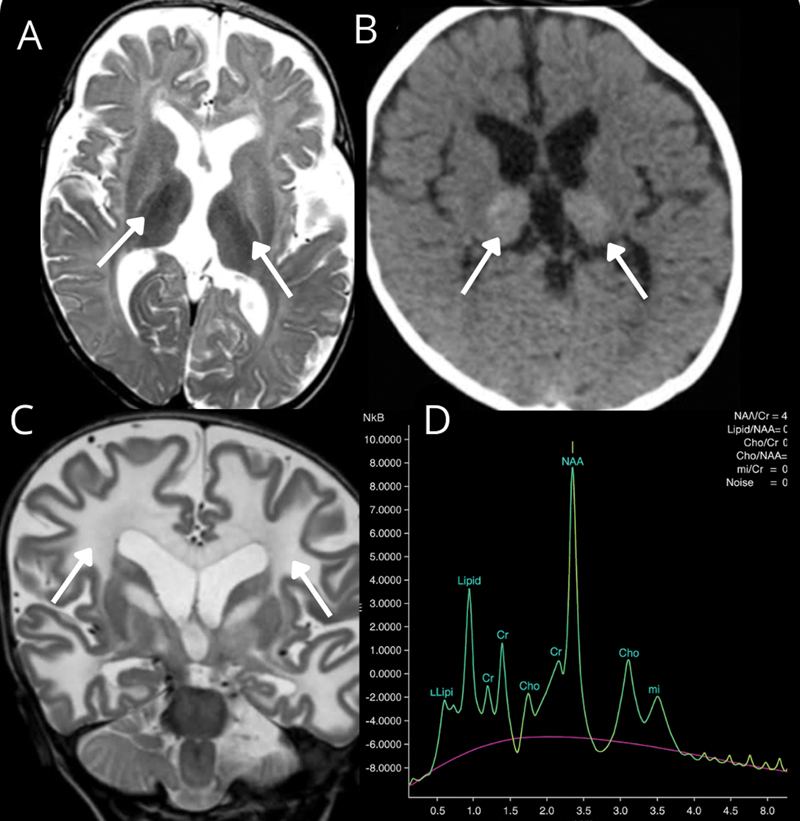
Abbreviations: CT, computed tomography; MRI, magnetic resonance imaging; MRS, MR spectroscopy; NAA, N-acetylaspartate. Krabbe and Canavan diseases: key neuroimaging findings (CT/MRI and MRS). ( **A** , **B** ) Krabbe disease (juvenile onset). ( **A** ) Axial T2-weighted MRI shows diffuse hyperintensity of the deep white matter. Thalamic hypointensity is also present (arrows). ( **B** ) Axial CT demonstrates bilateral thalamic hyperdensities. These findings are consistent with juvenile-onset Krabbe disease, which typically shows slowly progressive demyelination and optic pathway involvement. ( **C** , **D** ) Canavan disease. ( **C** ) Axial T2-weighted MRI demonstrates diffuse hyperintensity of the subcortical and deep white matter (arrows), with bilateral involvement of the globus pallidus. ( **D** ) MR spectroscopy shows an elevated NAA peak (arrow), a suggestive finding in Canavan disease.

In these cases, MRI is critical for early recognition. Also, T2 hyperintensity of the cerebellum (hilum of the dentate nucleus, surrounding low intensity of the peridentate area, and increased intensity of the surrounding cerebellar white matter) in early Krabbe's disease is a common feature. 35 Decreased intensity in the thalamus on T2, enlargement of the optic nerves and chiasm, as well as, rarely, enhancement of multiple cranial nerves can be observed ( Figure 3 ). Involvement of the periventricular white matter and the centrum semiovale can also be seen early. As in MLD, cranial nerve or caudal nerve root enhancement may occur in the earlier stages of the disease. Furthermore, T2 hyperintensities in the corpus callosum, parietooccipital white matter, and atrophy tend to occur later in the course of the disease.

The use of DWI scans may show hyperintensity along the progression line of the active demyelinating process in the early stage of the disease, due to myelin edema. In more advanced stages of the disease, there is increased diffusivity, indicating myelin loss leading to decreased anisotropy.

Also, CT can demonstrate thalamic and other deep-structure hyperdensities, producing a distinctive imaging signature ( Figure 3 ). 35 Diagnosis hinges on reduced galactocerebrosidase activity confirmed by GALC sequencing, and MRI helps distinguish Krabbe from MLD or ALD through its characteristic optic-nerve, deep white matter, and corticospinal involvement. 34 Early hematopoietic stem-cell transplantation in presymptomatic neonates, identified via newborn screening and imaging, can slow progression and preserve neurologic function. 36

### Canavan's disease

Canavan's disease is an autosomal-recessive leukodystrophy caused by biallelic ASPA mutations that abolish aspartoacylase activity, allowing NAA to accumulate and trigger intramyelinic edema, spongiform degeneration and failed myelin formation. 37 Infants, usually between 3 and 6 months, present with macrocephaly, axial hypotonia, poor head control, and developmental regression that later gives way to spasticity, seizures, and visual loss; most die in early childhood from relentless neurodegeneration. 37 38

Analysis of MRI is highly characteristic, showing bilateral, symmetric T2/FLAIR hyperintensity of subcortical white matter—most marked frontally and parietally—with early globus-pallidus and thalamic involvement and, unlike many leukodystrophies, no sparing of U-fibers or contrast enhancement ( Figure 3 ). 37 Regarding DWI, scans show hyperintensity within abnormal white matter and decreased ADC values, consistent with isotropically restricted water diffusion, probably due to myelin edema. A diagnostic hallmark is the strikingly elevated NAA peak on proton MRS, 39 often three- to five-fold above normal ( Figure 3 ). 37 Confirmation relies on raised NAA in urine, cerebrospinal fluid (CSF) or brain MRS, plus ASPA sequencing. Although no cure exists, gene-replacement and substrate-reduction strategies are under investigation. 38

### Leukoencephalopathy with brainstem and spinal cord involvement and lactate elevation (LBSL)

**Figure 4:**
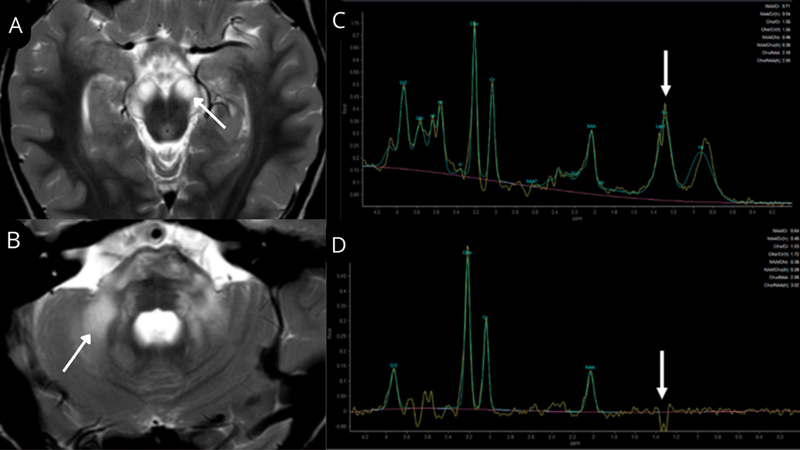
Abbreviations: MRI, magnetic resonance imaging; MRS, MR spectroscopy. Leukoencephalopathy with brainstem and spinal cord involvement and lactate peak on MRS. ( **A** , **B** ) Conventional MRI. Axial T2-weighted images at the level of the midbrain ( **A** ) and pons ( **B** ) show symmetric hyperintensities involving the corticospinal tracts (arrows) and middle cerebellar peduncles (arrows). ( **C** , **D** ) MRS of the brain demonstrates an elevated lactate peak: ( **C** ) TE 270 ms showing a lactate singlet at ∼1.3 ppm (arrow), and ( **D** ) TE 135 ms showing the characteristic inverted lactate doublet at ∼1.33 ppm (arrow).

Leukoencephalopathy with brainstem and spinal cord involvement and lactate elevation (LBSL) is caused by biallelic DARS2 mutations that disable mitochondrial aspartyl-tRNA synthetase, leading to tract-selective white matter injury. 40 41 42 The MRI scans typically show symmetric T2/FLAIR hyperintensity in periventricular cerebral white matter, together with signal abnormalities in the medullary pyramids, cerebral peduncles, cerebellar white matter, and the posterior and lateral corticospinal columns of the spinal cord. Meanwhile, proton MRS reveals a lactate peak that confirms mitochondrial dysfunction ( Figure 4 ). 42 43

As for DWI, scans show moderate hyperintensities within the involved deep white matter structures, which may reflect myelin edema. Measurement of mean diffusivity and fractional anisotropy may also indicate primary damage to the white matter structure.

Clinically, the disease begins in childhood or adolescence with slowly progressive cerebellar ataxia, spasticity, and dorsal-column proprioceptive loss; cognition is often relatively preserved, and many patients retain independent ambulation for years. 41 Diagnosis hinges on detecting pathogenic DARS2 variants, supplemented by elevated lactate in CSF or blood, and treatment is purely supportive, focusing on physiotherapy, orthopedic management, and seizure control, as no disease-modifying therapy is available. 41 43

### Vanishing white matter disease (VWM)

Vanishing white matter (VWM) disease, also called childhood ataxia with central nervous system hypomyelination, is one of the most frequent leukodystrophies. It is caused by autosomal recessive mutations in the EIF2B1 to EIF2B5 genes, which encode the subunits of the eIF2B complex, a key regulator of protein synthesis and the cellular stress response. 44 Dysfunction of this complex leads to selective vulnerability of astrocytes and oligodendrocytes, explaining the stress-provoked episodes of acute neurological deterioration seen in these patients. 45

Clinically, VWM typically presents in early childhood (2–6 years) with progressive cerebellar ataxia, spasticity, seizures, and cognitive decline. Ovarian failure in female patients is also well described, giving rise to the so-called “ovarioleukodystrophy” phenotype. 46 Earlier onset usually correlates with more rapid progression, consistent with lower residual eIF2B activity. 45

The MRI scans demonstrate a distinctive pattern: diffuse T2 hyperintensity of the white matter with progressive rarefaction and cystic degeneration, becoming isointense to CSF on all sequences. 44 The process often starts in periventricular and deep white matter, later extending to subcortical U-fibers, and is invariably associated with thinning and signal alteration of the corpus callosum. A characteristic “stripe-like” appearance on sagittal T1 corresponds to perivascular tissue remnants within areas of rarefaction. 44 There is no contrast enhancement or restricted diffusion; cystic regions show facilitated diffusion. Cerebellar and brainstem involvement may also be present, usually with atrophy and T2/FLAIR hyperintensity. 47

On DWIs, the cavitated regions are markedly hypointense, and other hyperintense areas moderately hypointense. These findings are consistent with a demyelinating process leading to myelin loss, and isotropically increased water diffusion.

### POLR3-related leukodystrophies

The POLR3-related leukodystrophy—formerly known as 4H syndrome—is an autosomal-recessive hypomyelinating disorder produced by pathogenic variants in POLR3A, POLR3B, and occasionally POLR1C or POLR3K, which disrupt RNA-polymerase-III function and impair oligodendrocyte maturation, leading to progressive loss of cerebral and cerebellar myelin. 48 Children present with motor delay, gait disturbance, cerebellar ataxia, and spasticity, while extraneurological signs, such as hypodontia, short stature and, later, hypogonadotropic hypogonadism provide clinical clues. 48 49

**Figure 5:**
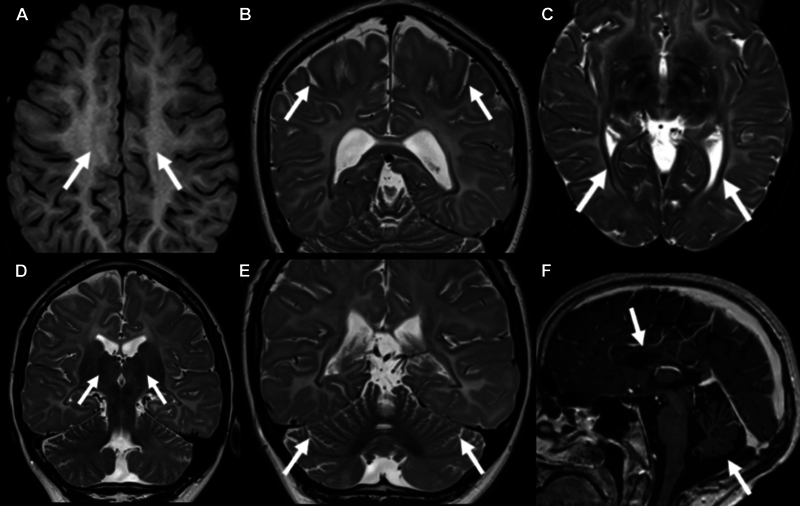
Magnetic resonance imaging (MRI) findings in POLR3-related leukodystrophies – diffuse hypomyelination, cerebellar atrophy, and thalamic T2 hypointensity. Axial T1-weighted ( **A** ) and axial T2-weighted ( **c** ), as well as coronal T2-weighted images ( **B,D,E** ) ( **A** – **E** ), demonstrate diffuse cerebral white matter hypomyelination, with relative preservation of the perirolandic region (arrows). ( **C** ) shows hypointensity of the optic radiation (arrows). ( **D** ) demonstrates bilateral anterolateral thalamic T2 hypointensity. ( **E** ) shows cerebellar atrophy (arrows). A postcontrast sagittal T1-weighted image ( **F** ) demonstrates thinning of the corpus callosum (superior arrow) and confirms cerebellar atrophy (inferior arrow).

When interpreted in the appropriate clinical context, MRI is highly informative, showing diffuse, symmetric T2/FLAIR hyperintensity of cerebral white matter and cerebellum, selective involvement of the posterior limbs of the internal capsule and corticospinal tracts, a thin but preserved corpus callosum, early vermian atrophy, and T2 hypointensity of the optic radiations and anterolateral thalami—all without contrast enhancement or inflammatory change ( Figure 5 ). 50 51 Confirmation requires sequencing of the relevant POLR3 subunit genes, as mutations can reside in any coding exon or splice site, and management remains supportive. 48

### Adult-onset leukoencephalopathy with axonal spheroids and pigmented glia (ALSP)

Adult-onset leukoencephalopathy with axonal spheroids and pigmented glia (ALSP) arises from heterozygous CSF1R mutations that impair microglial homeostasis, leading to axonal degeneration and inflammatory demyelination. 52

Typically, MRI reveals bilateral—but often asymmetric—T2/FLAIR hyperintensities in frontal and parietal white matter, early thinning and signal change of the corpus callosum, progressive frontal atrophy, and diffusion-restricted foci indicating active axonal injury. 53 The use of CT scans can show punctate calcifications in periventricular white matter or basal ganglia, and 1H-MRS may demonstrate reduced NAA with elevated choline, 53 all of which, together with the characteristic corpus-callosal involvement depicted in **Supplementary Material Figures S2** and **S3** , strongly suggest ALSP.

Clinically, the disease begins between 30 and 50 years with neuropsychiatric disturbances—apathy, depression, personality change, cognitive decline—followed by parkinsonism, spasticity, and gait impairment, progressing to severe disability or death within a decade. 52 54 Definitive diagnosis depends on identifying a pathogenic CSF1R variant, but when genetic testing is unavailable, the combination of imaging and clinical features supports a presumptive diagnosis; treatment is purely supportive.

### Glutaric acidurias (GA1 and L-2-hydroxyglutaric aciduria)

Glutaric aciduria (GA) is a cerebral organic aciduria caused by mitochondrial glutaryl-CoA dehydrogenase (GCDH, EC 1.3.8.6) enzyme deficiency. 55 56 In early diagnosed patients, the frequency of encephalopathic episodes and movement disorders was significantly reduced; current treatment strategies are based on levocarnitine, vitamin B2, and diet. 55 56 The neurological findings at the time of diagnosis can include seizures, developmental delay, intellectual disability, movement disorders, and tone abnormality. 55 56 The presence of L-2-hydroxyglutaric aciduria predominantly affects subcortical white matter. Periventricular white matter is spared. Globus pallidus and dentate nucleus hyperintensities may be observed on T2/FLAIR weighted images.

As for DWIs, scans demonstrate white matter lesions with hypointensity, consistent with significant myelin loss and resultant isotropically increased water diffusion. This finding corroborates loss of tissue matrix in the context of a very slowly progressive disease, which histologically corresponds to a spongiform encephalopathy. Proton MRS often shows an elevated choline peak and occasionally lactate, reflecting demyelination and metabolic stress. 55

### Neuronal ceroid lipofuscinoses (NCLs)

Neuronal ceroid lipofuscinoses (NCLs) represent a group of inherited lysosomal storage disorders characterized by progressive neurodegeneration, epilepsy, cognitive and motor decline, and visual impairment. 57 Although classified as inherited metabolic leukoencephalopathies rather than primary leukodystrophies, 58 they are often included in neuroradiological reviews of the latter because of reported white matter abnormalities on MRI. 59 They are genetically heterogeneous, with pathogenic variants in lysosomal enzymes such as PPT1 (CLN1) and TPP1 (CLN2), as well as proteins involved in lysosomal membrane function and trafficking. 57 The pathological hallmark is the accumulation of autofluorescent lipopigments within neurons and other cell types, reflecting incompletely degraded proteins and lipids stored in lysosomes. 60

The clinical manifestations vary with subtype and age of onset but share common elements of epilepsy, developmental regression, cognitive decline, and visual loss. Typically, CLN1 begins in the first 2 years with rapid neurodegeneration and seizures, followed by progressive visual decline that often culminates in blindness. As for CLN2, it usually presents between 2 and 4 years with language delay, seizures, and ataxia, followed by loss of motor and cognitive skills. The most common juvenile form, CLN3, begins in school age, with progressive visual failure as the earliest manifestation, preceding epilepsy and cognitive decline. Later–onset variants show slower progression but ultimately follow a similar degenerative course. 57

Brain MRI shows a combination of signal changes and atrophy. The presence of hypointensity of the thalami on T2/FLAIR is a consistent feature, present in most patients. Hyperintensity in periventricular and deep white matter, the posterior limb of the internal capsule, ventral pons, and insular/subinsular regions are frequently observed. Both supra- and infratentorial atrophy are common, and follow–up studies demonstrate progressive worsening over time. 61 These imaging features, in correlation with the clinical picture of epilepsy, regression, and visual decline, strongly support the diagnosis and help to distinguish NCLs from primary leukodystrophies. 59

### Kearns-Sayre syndrome (KSS)

The KSS is a rare sporadic mitochondrial disease caused by a single large-scale mitochondrial DNA (mtDNA) deletion. 5 The clinical manifestations occur before 20 years, and include progressive external ophthalmoplegia, pigmentary retinopathy, heart blocks, cerebellar ataxia, sensory hearing loss, short stature, and endocrinopathy. 5

Cerebral and cerebellar atrophy with bilateral and symmetric T2 hyperintensities in subcortical cerebellar and cerebral white matter, medio-dorsal thalamus, basal ganglia, corpus callosum, and dorsal brainstem are the most commonly reported brain MRI findings. 62 Subcortical U-fibers are mainly involved, while deep periventricular white matter is usually spared. Spinal cord involvement is common in KSS and may show different patterns, including white and gray matter. 63

In conclusion, the use of MRI scans, particularly when complemented by DWI and MRS, remains central to the early recognition and classification of leukodystrophies. By organizing disorders according to reproducible imaging patterns and regional predilection, this pictorial review provides a practical framework that can shorten diagnostic delay, support timely referral for disease-modifying options when available, and improve longitudinal monitoring. Furthermore, the case-based figures and summary diagrams provide an educational resource that reinforces a systematic, pattern-recognition approach, supporting training in neuroradiology and neurology and improvement of interdisciplinary communication.

## TAKEAWAYS

1. **Genetic basis as diagnostic pillar**
Leucodystrophies represent a heterogeneous group of genetically determined white matter disorders. Mutations in genes encoding enzymes ( *ARSA* , *GALC* , *ASPA* ), structural proteins ( *GFAP* ), or mitochondrial machinery ( *DARS2* , *L2HGDH* ) underlie distinct pathophysiological mechanisms, from lysosomal storage defects to mitochondrial translation failure. Understanding the molecular etiology is crucial not only for diagnosis, but also for emerging precision therapies.
2. **MRI as a key diagnostic tool**
  The use of MRI remains the cornerstone of diagnosis, revealing characteristic patterns that can guide the differential diagnosis, such as:
  - Radial streaks and relative U-fiber sparing (often seen in MLD, but not specific).
  - Trizonal demyelination with a contrast-enhancing rim in ALD.
  - Frontal predominance in Alexander's disease and in the frontal variant of ALD.
  - Tract-selective signal abnormalities in LBSL and Krabbe.
  Accurate interpretation of MRI, combined with MRS when available (e.g., elevated NAA in Canavan disease or lactate in LBSL), can substantially narrow the differential diagnosis.
3. **Pathophysiology drives imaging**
  The imaging findings are not merely descriptive, but reflect underlying biochemical derangements:
  - Sulfatide accumulation (MLD) → radial hypointensities;
  - Psychosine toxicity (Krabbe) → corticospinal degeneration;
  - Defective tRNA synthetase (LBSL) → mitochondrial tractopathy.
  Linking imaging to molecular dysfunction enables a mechanistic understanding essential for early recognition and research development.
4. **White matter selectivity matters**
  A predilection for specific white matter regions may be depicted in the disorders mentioned in this manuscript:
  - Frontal lobes (Alexander's disease, ALSP, frontal ALD)
  - Parieto-occipital regions (ALD)
  - Corticospinal and posterior columns (Krabbe, LBSL)
  Recognizing these selective vulnerabilities can guide genetic testing and avoid diagnostic delays.
5. **Early diagnosis is therapeutically meaningful**
  Although curative therapies remain limited, timely recognition allows for:
  - Early HSCT in ALD and Krabbe, improving prognosis if initiated pre-symptomatically.
  - Clinical trial enrollment (e.g., gene therapy in Canavan or POLR3-LDs).
  - Genetic counseling and multidisciplinary support, especially in progressive or multisystemic cases.
6. **Integration is key**
  Optimal diagnostic accuracy arises from the integration of clinical signs, MRI phenotypes, MRS (when applicable), and genetic confirmation. Standalone interpretation, whether clinical or radiological, is often insufficient in most cases.
